# Caulis Spatholobi extracts inhibit osteosarcoma growth and metastasis through suppression of CXCR4/PI3K/AKT signaling

**DOI:** 10.1186/s13018-023-04437-6

**Published:** 2023-12-21

**Authors:** Yang Jiang, Yemei Gao, Xin Li, Fangming He, Yang Liu, Renxian Wang

**Affiliations:** 1 Traditional Medicine Department, Beijing Jishuitan Hospital, Capital Medical University, Beijing, China; 2 Laboratory of Bone Tissue Engineering, Beijing Laboratory of Biomedical Materials, Beijing Research Institute of Traumatology and Orthopaedics, Beijing Jishuitan Hospital, 31 Xinjiekou East Street, Xicheng District, Beijing, 100035 China

**Keywords:** Caulis Spatholobi, CXCR4, PI3K/AKT axis, Osteosarcoma, Metastasis

## Abstract

**Background:**

The therapeutic potential of Caulis Spatholobi (CS) extracts against various cancers has been well documented, yet its impact and mechanism in osteosarcoma (OS) remain unexplored. This study aims to elucidate the effects of CS extracts on the growth and metastasis of OS, along with its underlying molecular mechanism.

**Methods:**

The impact of CS extracts on the proliferative potential of two OS cell lines (Saos-2 and U2OS) was assessed using MTT and colony-formation assays. Additionally, the migratory and invasive capacities of OS cells were investigated through Transwell assays. The modulation of CXCR4 expression by CS extracts was evaluated using qRT-PCR and Western blotting. Furthermore, the influence of CS extracts on the activation of PI3K/Akt signaling was determined through Western blotting.

**Results:**

CS extracts exhibited a dose- and time-dependent inhibition of proliferation and colony formation in OS cells. Notably, CXCR4 expression was prominently observed in Saos-2 and U2OS, and treatment with CS extracts led to a dose-dependently reduction in CXCR4 levels. Silencing CXCR4 or inhibiting its function diminished the migratory and invasive capacities of OS cells. Conversely, the CS extracts induced suppression of OS cell migration and invasion was counteracted by CXCR4 overexpression. Mechanistically, CS extracts repressed PI3K/AKT signaling in OS cells by downregulating CXCR4 expression.

**Conclusions:**

CS extracts mitigate the CXCR4/PI3K/AKT signaling-mediated growth and metastasis capacities of OS cells, thus might play an anti-tumor role in OS.

## Background

Osteosarcoma (OS) is a prevalent primary bone tumor, also known as osteogenic sarcoma due to its origin from osteogenic mesenchymal tissue [1]. It predominantly affects teenagers and children under 20 years of age [2]. OS tumors exhibit characteristics of high malignancy, early metastasis and poor prognosis, as they are rich in blood vessels and easily spread through the bloodstream. The current clinical approach for treating osteosarcoma involves a combination of neoadjuvant chemotherapy and surgical resection. Chemotherapy drugs commonly used include doxorubicin, cisplatin, ifosfamide, and methotrexate, etc. Despite the significant efficacy of this combined treatment, studies have shown that [3, 4] more than 40% of OS patients eventually develop distant metastases in the lungs and bones, with lung metastases accounting for 90% of these cases. Approximately, 13 to 27 percent of OS patients are diagnosed with pulmonary metastases initially [5], which is accompanied by a grim prognosis and a 5-year survival rate of only 20–29% [6]. Therefore, exploring the molecular mechanisms underlying OS progression and metastasis is crucial for identifying novel and effective therapeutic targets and strategies.

CXC-chemokine receptor 4 (CXCR4) is a receptor for stromal cell-derived factor-1 (SDF-1), which is primarily secreted by stromal cells such as fibroblasts and endothelial cells. It is widely expressed in stromal cells of various organs and tissues, including blood vessels. Research has shown that CXCR4 was overexpressed in various tumor cells, such as breast and prostate cancers. It has been demonstrated to interact with SDF-1, inducing chemotactic migration of target cells and enhancing cell adhesion, thereby promoting tumor invasiveness and metastasis [6]. Recent studies have revealed [7] that CXCR4 is critical in driving, guiding and locating OS metastasis in the bone marrow and lungs. Moreover, CXCR4 may influence multiple signaling pathways such as PI3K/AKT, ERK1/2, JNK and c-Jun to modulate tumor progression. Among these pathways, the PI3K/AKT axis has been confirmed to play a vital role in the invasion and metastasis of various solid tumors [8, 9]. However, further investigation is required to elucidate the specific molecular mechanisms by which CXCR4 influences OS growth and metastasis.

In traditional medicine, Caulis Spatholobi (CS) is a renowned Chinese herbal medicine with anti-aggregation effects, which are widely utilized in disease treatment to improve blood circulation and eliminate blood stasis. Previous studies have demonstrated the anti-metastatic activity of CS in tumors [10]. In mouse models bearing tumors, CS extracts significantly inhibited lung metastasis of breast cancer cell lines and prolonged the survival period of mice. It has also reported that CS extracts can exert anti-tumor metastasis effects by blocking platelet aggregation induced by tumor cells [11]. In this study, we investigated the effects of CS extracts on OS cell proliferation, migration and invasiveness. We further explored whether these extracts could inhibit OS growth and metastasis by targeting CXCR4 and regulating PI3K/AKT axis, aiming to provide a novel strategy for OS.

## Methods

### Cell cultivation

Human osteoblasts hFOB1.19, OS Saos2 and U2OS cells were procured from American Type Culture Collection (VA, USA). The cells were cultivated in DMEM (Thermo Fisher Scientific, Massachusetts, USA) supplemented with 10%FBS and 100U/mL penicillin streptomycin. The hFOB1.19 cells were maintained at 33 °C, while Saos2 and U2OS were cultured at 37 °C in a 5%CO_2_ incubator.

### CS extraction

Fifteen gram of sieved (20 mesh) CS powder was mixed with water at a ratio of 1:10 (weight to volume) as the extraction solvent and was subjected to two rounds of hot reflux extraction for 1 h each. After filtration, the filtrates were combined. Subsequently, the CS extracts were concentrated using a rotary evaporator and a vacuum oven until dry. The resulting weighted CS extracts were dissolved in DMSO to obtain a solution with a concentration of 2 mg/mL.

### Cell treatment and transfection

To knock out CXCR4, Saos2 and U2OS were treated with DMEM and a 10-µM CXCR4-specific antagonist AMD3100 (Sigma-Aldrich) or a synthetic small interfering RNA targeting CXCR4 (si-CXCR4) for 24 h. The si-CXCR4 was synthesized by Gene Tech (Shanghai, China), with non-targeting small interfering RNA (siRNA) serving as a negative control (si-NC). The human CXCR4 sequence was then inserted into the pcDNA3.1 plasmid (GenePharma) to create oe-CXCR4. The empty pcDNA3-1 plasmid was used as a negative control (oe-NC). Cell transfection was carried out by Lipofectamine 3000 following manufacturer's instructions. The transfection efficiency of CXCR4 plasmid and siRNA was determined 48 h later using RT-qPCR and western blotting.

### MTT

Saos2 and U2OS were seeded in 96-well plates at a density of 4 × 10^3^ cells/well and treated with various concentrations of CS (50, 100, 200, 400 and 800 μg/mL) for 24, 48 and 72 h. The MTT assay was performed to assess OS cell viability after drug treatment. Following a 4-h incubation with MTT solution (Sigma-Aldrich, MO, USA),absorbance at 570 nm was measured using a microplate reader from Molecular Devices (CA, USA). The half-maximum inhibitory concentration (IC_50_) of CS at each time point was calculated using GraphPad Prism software (v8.0; GraphPad software, Inc., CA, USA).

### Colony formation assay

Saos2 and U2OS were seeded in 6-well plates at a density of 200 cells/well and incubated overnight for cell adhesion. Subsequently, they were exposed to various concentrations of CS (50, 100, and 200 μg/mL) and cultured for 14 days. The colonies were fixed with 4% paraformaldehyde for 30 min, stained with 0.1% crystal violet for 10 min, then photographed and counted under a microscope (Olympus, Tokyo, Japan).

### RT-qPCR

Total RNA from Saos2 and U2OS cells was isolated using TRIzol reagents (Thermo Fisher Scientific) and subjected to first-strand cDNA synthesis using a PrimeScript™ RT kit (Beijing Tiangen, China). RT-qPCR analysis was performed on a real-time PCR system (Bio-Rad, California, USA) with a SYBR®Green Master Mix (TaKaRA, Japan) to detect the mRNA expression of CXCR4, PI3K, Akt, and other target genes, using β-actin as an internal reference. The relative expression of the target gene was quantified by calculating the ratio of the Ct value of the target gene to that of the internal reference gene.

### Western blotting

Total proteins were extracted from Saos2 and U2OS cells using RIPA lysis buffer (Solarbio, Beijing, China) containing a protease inhibitor mixture (Sigma-Aldrich). After quantification using a BCA kit (Beyotime, Shanghai, China), the proteins were separated by SDS-PAGE and transferred to a PVDF membrane. Subsequently, the membrane was incubated with primary and secondary antibodies, and the protein signals were captured using a chemiluminescence device (Tanon, Shanghai, China) and quantified by Image J software.

### Transwell migration and invasiveness analyses

For cell migration evaluation, Saos2 and U2OS cells (2 × 10^5^ cells/well) were collected and resuspended in 200 μL FBS-free medium and placed in the apical chamber of a 24-well Transwell culture chamber (Corning, New York, USA) after designated treatment or/and transfection. The basolateral chamber was filled with 750 μL of 10% FBS-supplemented DMEM medium. After 24 h of incubation, migrated cells were fixed in the basolateral chamber with 4% paraformaldehyde for 30 min, and stained with 0.1% crystal violet for 20 min. Finally, the migrated cells were photographed by microscope (Olympus), and the stained cells were counted by Image J software. For cell invasiveness assessment, the Transwell chamber was precoated with Matrigel (Sigma-Aldrich), with subsequent procedures similar to those for migration detection.

### Statistical methods

All the experiments were repeated at least three times. Statistical analysis was performed using GraphPad Prism v5.0 (GraphPad Software, La Jolla, CA, USA), and SPSS Statistics 21.0. Data were presented as mean ± standard deviation (SD). Statistical significance was determined by Student's t-test or one-way analysis of variance followed by LSD post hoc test with a significance level set at *P* < 0.05.

## Results

### CS extracts suppress OS cell growth

**Fig. 1:**
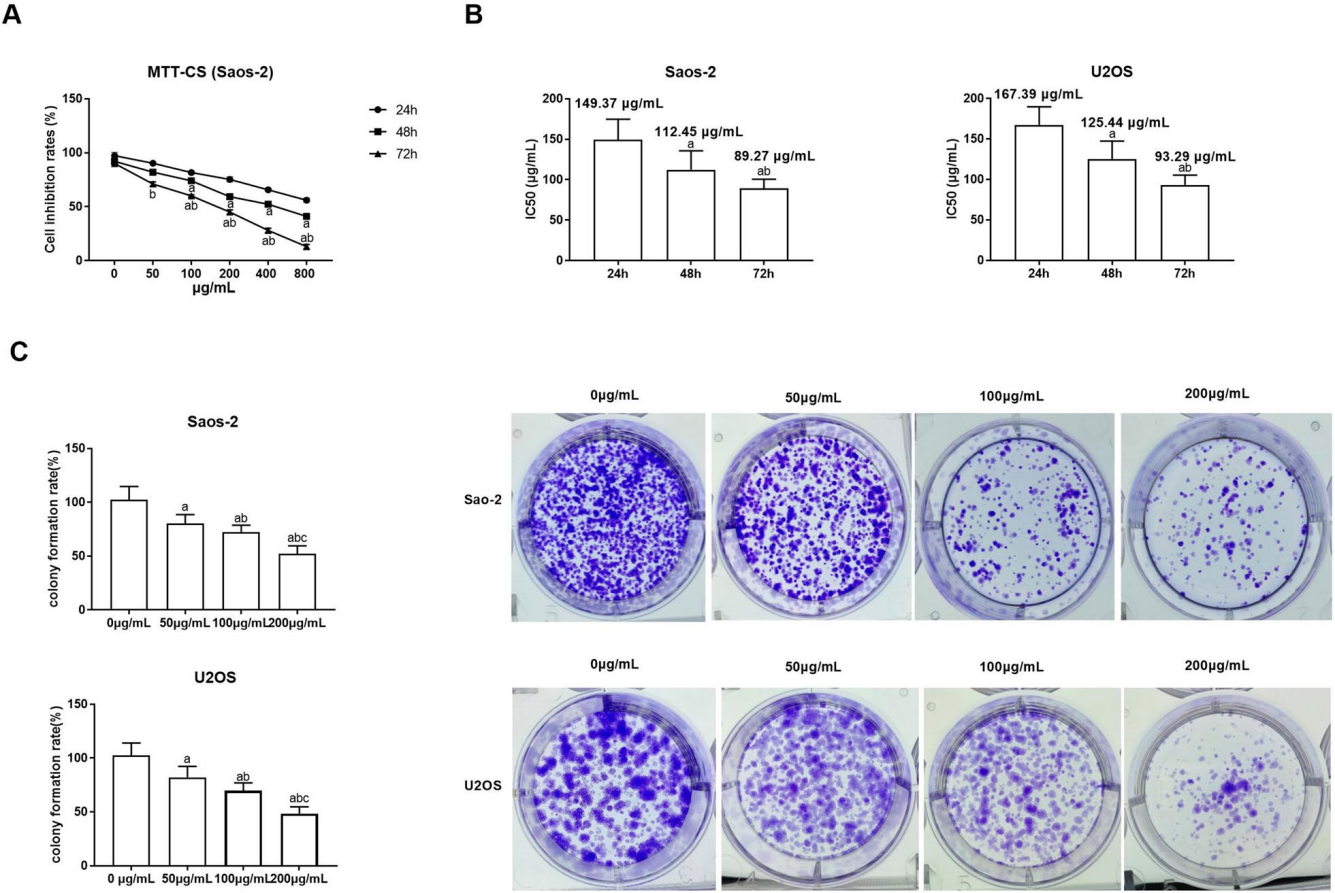
CS inhibits OS cell proliferation. **A** Suppression rates of Saos-2 were ascertained via MTT assay subsequent to exposure to 50, 100, 200, 400, and 800 µg/mL CS for 24, 48, and 72 h. **B** The half-maximum inhibitory concentration (IC_50_) was gauged at 24, 48, and 72 h. In comparison with the 24-h group, ^a^*P* < 0.05; compared with 48 h group, ^b^*P* < 0.05. **C** Saos-2 and U2OS cell proliferation following treatment with 0, 50, 100, and 200 µg/mL CS was detected by colony formation assay. Compared with 0 µg/mL group, ^a^*P* < 0.05; Compared with 50 µg/mL group, ^b^*P* < 0.05; compared with 100 µg/mL group, ^c^*P* < 0.05. N = 3; data are presented as the mean ± standard deviation. CS: Caulis Spatholobi

Initially, the viability of Saos-2 and U2OS cells was assessed using the MTT assay (Fig. 1A) following exposure to varying concentrations (0, 50, 100, 200, 400, and 800 µg/mL) of CS extracts for 24, 48, and 72 h to investigate the impact of CS extracts on OS growth. It was observed that with prolonged culture duration, the inhibition rate of Saos-2 and U2OS cells increased (*P* < 0.05). Concurrently, as the concentration of CS extract increased, the cell survival rate decreased over time (*P* < 0.05). The half maximal inhibitory concentration (IC_50_), a critical indicator for drug evaluation representing the concentration at which a substance inhibits certain biological processes by 50%. IC_50_ is usually used to measure the toxicity of drugs to cells or the tolerance of cells to drugs. The IC_50_ decreased over time for Saos-2 and U2OS cells (*P* < 0.05), as shown in Fig. 1B. Following treatment with 50, 100, and 200 µg/mL CS extracts, the colony formation rate of Saos-2 and U2OS cells, as determined by the colony-formation assay, significantly decreased with escalating CS extract concentration (*P* < 0.05), as shown in Fig. 1C.

### CS extracts reduce CXCR4 expression in OS cells

**Fig. 2:**
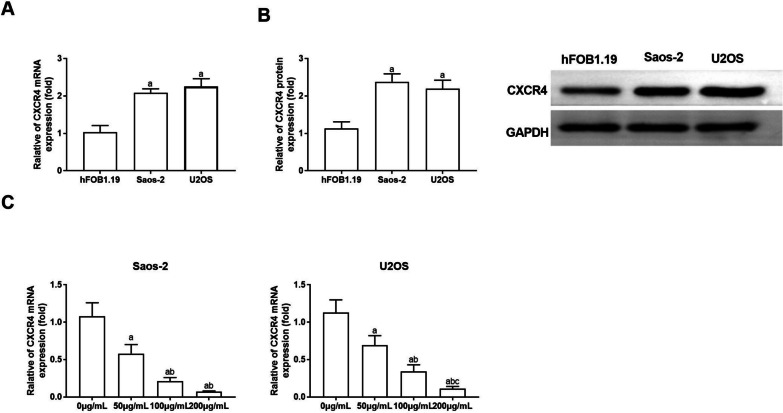
CS diminishes CXCR4 expression in OS cells. The mRNA and protein expression of CXCR4 in hFOB1.19, Saos-2 and U2OS were assessed using RT-qPCR (**A**) and Western blotting (**B**), respectively. Compared with hFOB1.19 group, ^a^*P* < 0.05. (C) RT-qPCR quantified CXCR4 expression in Saos-2 and U2OS treated with 50, 100 and 200 µg/mL CS for 24 h. Compared with 0 µg/mL group, ^a^*P* < 0.05; Compared with 50 µg/mL group, ^b^*P* < 0.05; compared with 100 µg/mL group, ^c^*P* < 0.05. *N* = 3; the results are described as the mean ± SD. CS: Caulis Spatholobi

Subsequently, we investigated whether the impact of CS extracts on OS progression was associated with CXCR4 expression by assessing CXCR4 expression at both mRNA and protein levels in human normal osteoblasts hFOB1.19, OS Saos-2 and U2OS cells using RT-qPCR and WB. CXCR4 transcription and protein levels were notably higher in Saos-2 and U2OS compared to hFOB1.19 (*P* < 0.05), as shown in Fig. 2A, B. Upon treatment with 0, 50, 100 and 200 µg/mL CS extracts, the CXCR4 transcription level markedly decreased with increasing CS extract concentration (*P* < 0.05), as shown in Fig. 2C.

### Blocking CXCR4 expression suppresses OS cell migration and invasiveness

**Fig. 3:**
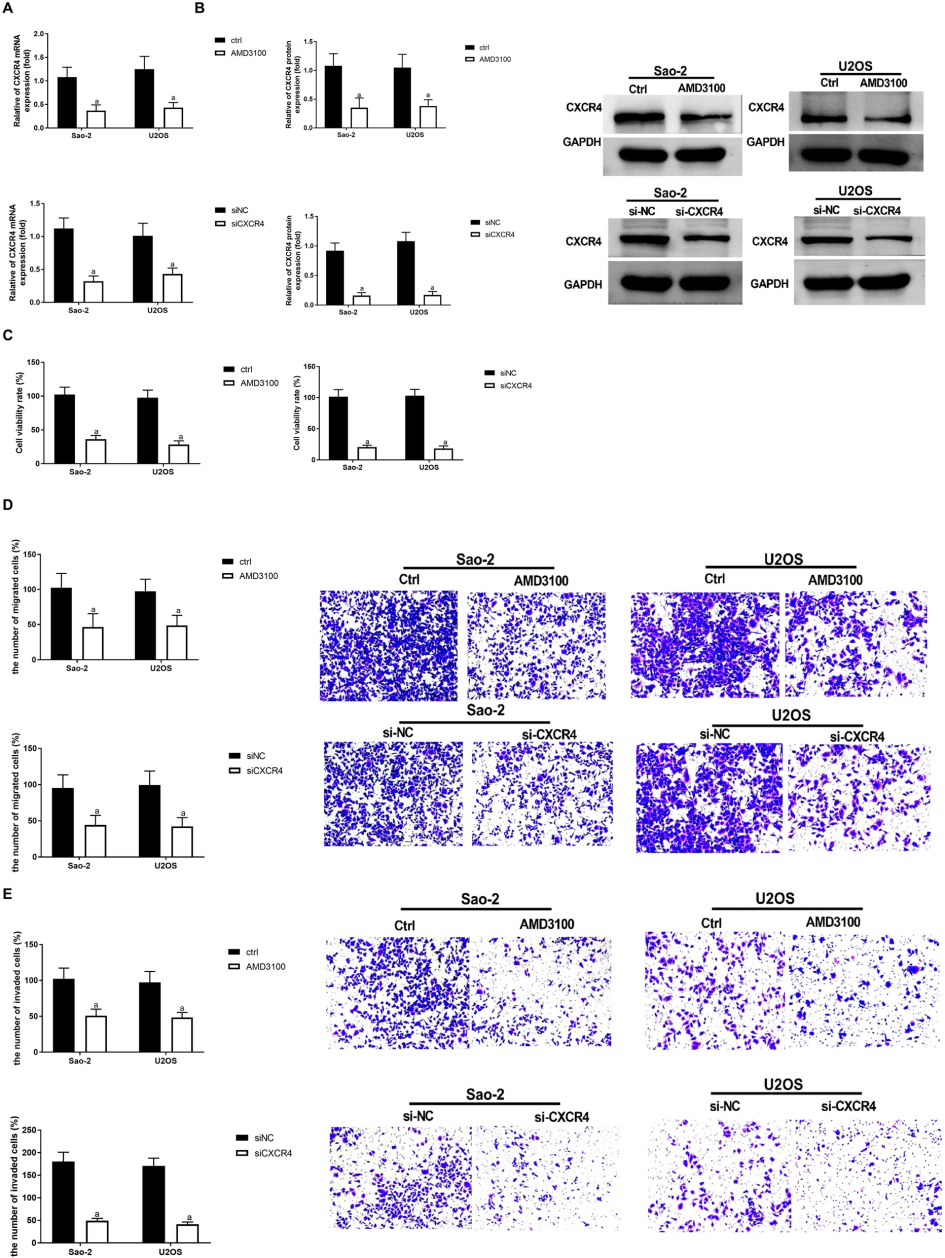
CXCR4 knockout suppresses OS cell migration and invasiveness. RT-qPCR **A** and **B** Western blotting determined CXCR4 expression in Saos-2 and U2OS treated with AMD3100 or transfected with si-CXCR4. (C) MTT assay detected Saos-2 and U2OS cell viability after AMD3100 treatment or si-CXCR4 transfection. **D**–**E** Transwell analysis was performed to observe the migration and invasiveness of Saos-2 and U2OS treated with AMD3100 or transfected with si-CXCR4. *N* = 3; the results are represented by the mean ± standard deviation. In comparison with the control group or si-NC group, ^a^*P* < 0.05. CS, Caulis Spatholobi

To investigate the potential relationship between the inhibition of cell growth and metastasis in OS and the inhibition of CXCR4, we initially employed AMD3100 and CXCR4 siRNA, which are small molecule inhibitors targeting CXCR4, to assess the impact of CXCR4 inhibition on OS cell migration and invasion. Through RT-qPCR and Western blot analysis, it was observed that Saos-2 and U2OS cells treated with AMD3100 or transfected with si-CXCR4 exhibited significantly reduced levels of CXCR4 transcription and protein levels compared to Ctrl or siNC negative control groups (*P* < 0.05), as shown in Fig. 3A, B. Furthermore, MTT and Transwell assays results demonstrated a substantial decrease in cell viability, migration, and invasiveness in Saos-2 and U2OS cells following AMD3100 treatment or si-CXCR4 transfection (*P* < 0.05), as shown in Fig. 3C–E.

### CS extracts inhibit OS cell migration and invasiveness by targeting CXCR4

**Fig. 4:**
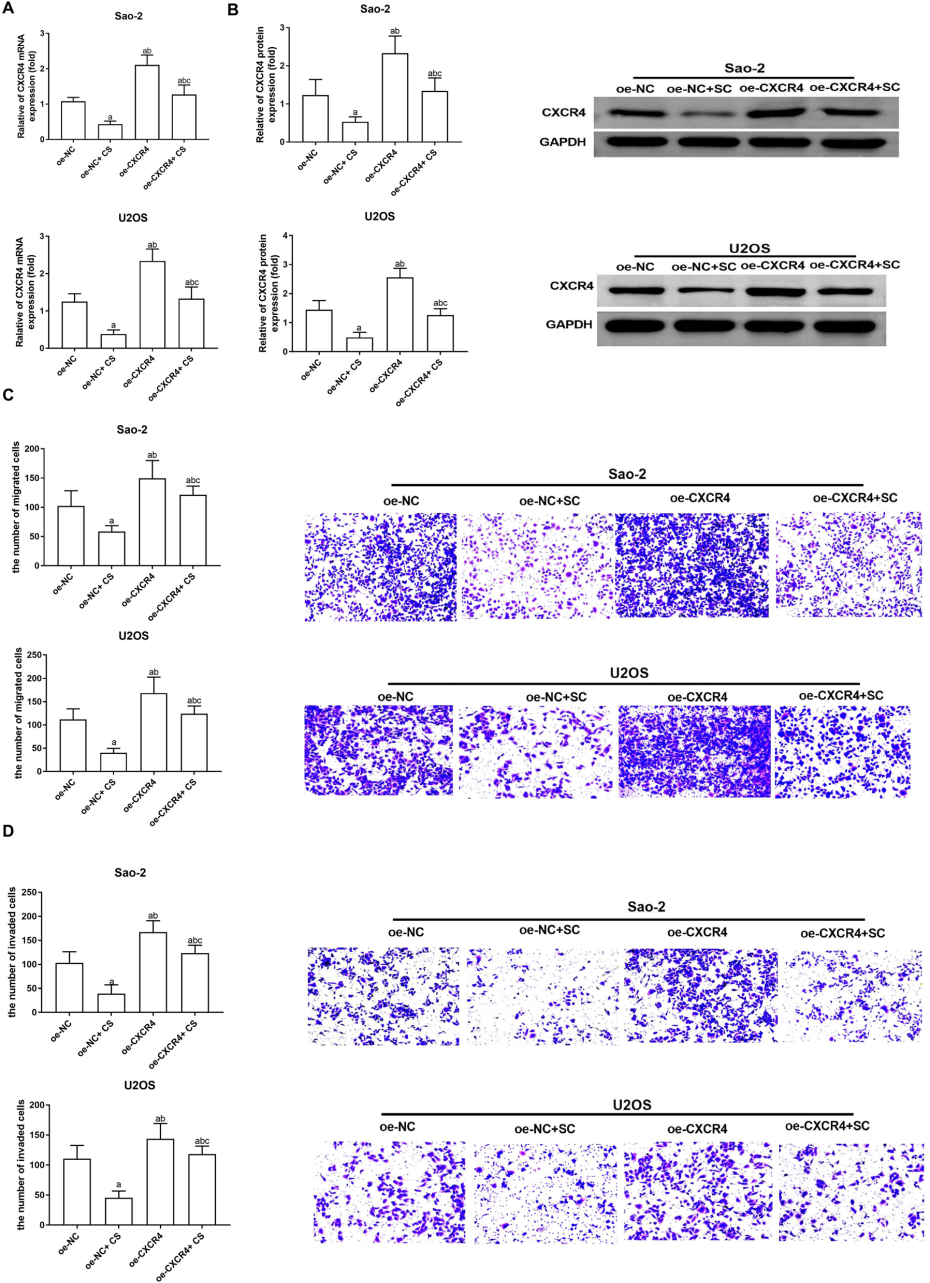
CS attenuates OS cell migration and invasiveness by downregulating CXCR4. Saos-2 and U2OS were transfected with oe-CXCR4, and then treated with 200 μg/mL CS for 24 h. RT-qPCR (**A**) and Western blotting (**B**) quantified CXCR4 mRNA and protein expression, respectively. **C**, **D** Transwell assay tested cell migration and invasiveness. *N* = 3; the results are expressed as the mean ± standard deviation. Compared with oe-NC group, ^a^*P* < 0.05; compared with oe-NC + CS group, ^b^*P* < 0.05; compared with oe-CXCR4 + CS group, ^c^*P* < 0.05. CS: Caulis Spatholobi

To further confirm whether the inhibition of OS cell growth and metastasis by CS is directly achieved through CXCR4 inhibition, we conducted validation using both CS extract therapy and CXCR4 overexpression. As shown in Fig. 4, Saos-2 and U2OS cells transfected with oe-CXCR4 exhibited higher CXCR4 transcription and protein expression levels as well as increased migration and invasiveness compared to the oe-NC group (*P* < 0.05). In contrast, CXCR4 transcription and protein expression in Saos-2 and U2OS cells in the oe-NC + CS group significantly decreased after 24 h of treatment with 200 μg/mL CS extracts, and the levels of cell migration and invasiveness were notably inhibited (*P* < 0.05). Moreover, compared with the oe-NC + CS group, CXCR4 transcription and protein levels in the oe-CXCR4 + CS group were elevated, and the cell migration and invasiveness capacity were enhanced (*P* < 0.05). It is suggested that the inhibition of CS extracts on CXCR4 transcription and protein expression as well as cell migration and invasiveness in Saos-2 and U2OS cells can be counteracted by overexpression of CXCR4.

### Inhibition of CXCR4 by CS extracts promote PI3K/AKT signal inactivation in OS cells

**Fig. 5:**
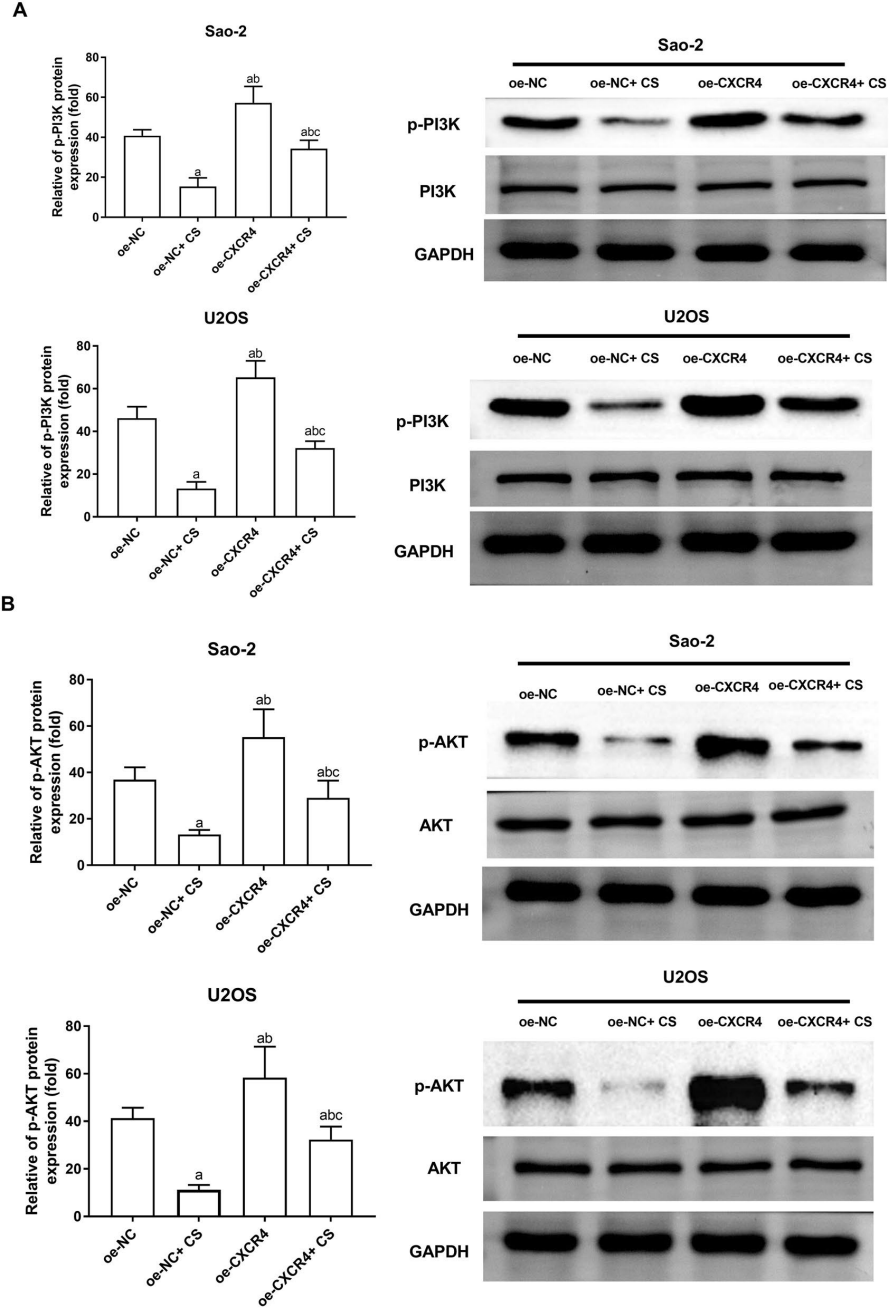
CS deactivates PI3K/AKT axis by down-regulating CXCR4. **A**, **B** Western blotting analysis was employed to measure p-PI3K, and p-AKT protein levels in Saos-2 and U2OS induced by CS (200 μg/mL, 24 h) or/and transfected by oe-CXCR4. *N* = 3; the data are calculated as the mean ± standard deviation. Compared with oe-NC group, ^a^*P* < 0.05; compared with oe-NC + CS group, ^b^*P* < 0.05; compared with oe-CXCR4 + CS group, ^c^*P* < 0.05. CS: Caulis Spatholobi; p: phosphorylated

Finally, we investigated the specific molecular mechanism through which CS extracts inhibit OS by targeting CXCR4. As shown in Fig. 5, p-PI3K and p-AKT protein levels in Saos-2 and U2OS cells were markedly higher in the oe-CXCR4 group and notably lower in the oe-NC + CS group when compared to the oe-NC group (*P* < 0.05). The oe-CXCR4 + CS group exhibited markedly higher p-PI3K and p-AKT protein levels than the oe-NC + CS group (*P* < 0.05). It is suggested that CS extracts can inhibit the p-PI3K/p-AKT axis in Saos-2 and U2OS cells by targeting CXCR4.

## Discussion

CS has favorable demonstrates potent inhibitory activity against various cancer cells and possesses anti-tumor properties. Its complex chemical composition and diverse pharmacological effects primarily consist of flavonoids, triterpenes, sterols, and anthraquinone compounds, with flavonoids, including isoflavones, dihydroflavonoids, and chalcones, being the most abundant. Previous studies have reported [12] that flavonoids in CS extracts effectively inhibited the proliferation and growth of human lung carcinoma, colorectal cancer and other tumor cells. Liu B et al. [13] also observed the inhibitory effect of various solvents extracts of CS on the growth of human OS Saos-2 cells, aligning with our own findings. In this study, through MTT assay results, revealed that the inhibition rates of Saos-2 and U2OS increased proportionally with the concentration of CS extract and duration of treatment. Moreover, the IC_50_ of CS extracts for OS cells decreased over time. Furthermore, research on CS extracts [14] have also documented their capacity to impede tumor activity, influence the cell cycle, and suppress tumor metastasis in transplanted Lewis lung cancer mouse models. Clinical pharmacological investigations [15] have highlighted the anti-thrombotic effects of CS in addition to its anticancer properties, with reported inhibition of induced metastasis [16]. CS extracts have been shown to effectively inhibit colorectal cancer invasion and metastasis by inhibiting ADP-induced platelet aggregation in vivo and in vitro, thereby further validating the impact of CS extracts on OS metastasis and invasiveness. This study demonstrated that CS extracts effectively reduced the abnormally high expression of CXCR4 in OS cells and effectively suppressed Saos-2 and U2OS migration, and invasiveness.

The metastasis of OS encompasses a diverse array of biological processes, including oncogenes and cell invasiveness and metastasis. Circulating tumor cells (CTCs) detached from the primary tumor site and adhered to the local migration matrix via stromal tissue, which is closely associated with cell signaling and cell–matrix interactions [17, 18]. CXCR4, a molecule known to be upregulated in a variety of cancers, hampers cancer cell angiogenesis and metastasis [19, 20]. In vitro studies have shown that CXCR4 inhibitors induce apoptosis and inhibit proliferation of OS cells [21]. Oda et al. [22] observed high expression of CXCR4 in metastatic and primary sites, which correlated with the activation of the vascular endothelial growth factor signaling pathway. This study revealed that CXCR4 was highly expressed in OS cells and inhibiting CXCR4 through inhibitors or CXCR4 gene knockout effectively impeded OS cell growth and metastasis. Additionally, CS extracts have shown efficacy in inhibiting OS cell migration and invasiveness by specifically regulating CXCR4. However, the precise mechanism by which CXCR4 modulates OS cell metastasis requires further elucidation, particularly regarding downstream signaling pathways. Tumor invasiveness and metastasis involve a network of interconnected signaling pathways, among which the PI3K/AKT and ERK1/2 axis have been demonstrated to play a pivotal role in the invasiveness and metastasis of solid tumors. Leelawat et al. [23] were the first to discover that the interaction between CXCR4 and chemokine SDF-1 could activate ERK1/2 and PI3K axis, inducing invasiveness in cholangiocarcinoma cells. Jiang et al. [24] showed that interfering with CXCR4 expression using siRNA, induced apoptosis in human OS cells, and the down-regulation of CXCR4 was achieved by inhibiting PI3K/Akt/NF-κβ axis, highlighting the significance of CXCR4 in the clinical treatment of OS. Further study revealed that, CXCR4/SDF-1 stimulated the phosphorylation of Akt, JNK and c-Jun, while the CXCR4 inhibitor AMD3100 reduced the phosphorylation activity in OS cells [25]. This study provided the first findings demonstrating that SDF-1 promoted the survival and metastasis of CXCR4-mediated OS by activating the Akt and JNK axis. AMD3100 counteracted the effects and downstream pathways influenced by CXCL12/CXCR4 interactions in the regulation of OS survival and metastasis. In recent times, an increasing number of CXCR4 inhibitors have entered clinical trials, demonstrating their pivotal role in treating patients unresponsive to checkpoint inhibitors, or those with cancer metastasis or recurrence. For instance, mavorixafor, an orally bioavailable CXCR4 antagonist, has shown promise in modulating immune cell trafficking in melanoma patients [26]. Balixafortide (BLX), a protein epitope mimetic inhibitor of CXCR4, has been shown to enhance docetaxel-mediated antitumor activity in PCa bone metastases [27]. Additionally, a novel CXCR4 inhibitor, modified with a picolinamide scaffold (CPZ1344), has entered pre-clinical research, demonstrating potential as a novel therapeutic agent against glioblastoma [28].

Our research demonstrates that CS extracts effectively inhibit the PI3K/AKT signaling pathway in OS cells, and overexpressing CXCR4 effectively prevents the inactivation of the PI3K/AKT signal caused by CS extracts in OS cells. Therefore, the impact of CS extracts on OS growth and metastasis is achieved by targeting CXCR4 to regulate the PI3K/AKT axis.

## Conclusions

In summary, this study reveals the involvement of CS in the growth and metastasis of OS and highlights how this traditional drug affects the proliferation, metastasis, and invasiveness of OS cells through the targeted regulation of the CXCR4/PI3K/AKT axis. This fundamental research suggests that CS holds potential as an effective drug for OS treatment, but further confirmation is warranted through animal experiments and clinical trials.

## Limitations

Considering that this investigation solely arrived at this deduction within in vitro cellular models, the conclusiveness of the outcomes necessitates further substantiation through in vivo experimentation.

## Data Availability

The data that support the findings of this study are available from the corresponding author, upon reasonable request.
